# Expression of Five Endopolygalacturonase Genes and Demonstration that *MfPG1* Overexpression Diminishes Virulence in the Brown Rot Pathogen *Monilinia fructicola*

**DOI:** 10.1371/journal.pone.0132012

**Published:** 2015-06-29

**Authors:** Chien-Ming Chou, Fang-Yi Yu, Pei-Ling Yu, Jia-Fang Ho, Richard M. Bostock, Kuang-Ren Chung, Jenn-Wen Huang, Miin-Huey Lee

**Affiliations:** 1 Department of Plant Pathology, National Chung-Hsing University, Taichung, Taiwan; 2 NCHU-UCD Plant and Food Biotechnology Center, National Chung-Hsing University, Taichung, Taiwan; 3 Agricultural Biotechnology Center, National Chung-Hsing University, Taichung, Taiwan; 4 Department of Plant Pathology, University of California, Davis, California, United States of America; Fujian Agriculture and Forestry University, CHINA

## Abstract

*Monilinia fructicola* is a devastating pathogen on stone fruits, causing blossom blight and fruit rot. Little is known about pathogenic mechanisms in *M*. *fructicola* and related *Monilinia* species. In this study, five endopolygalacturonase (endo-PG) genes were cloned and functionally characterized in *M*. *fructicola*. Quantitative reverse-transcriptase PCR (qRT-PCR) revealed that the five *MfPG* genes are differentially expressed during pathogenesis and in culture under various pH regimes and carbon and nitrogen sources. *MfPG1* encodes the major endo-PG and is expressed to significantly higher levels compared to the other four *MfPG*s in culture and *in planta*. *MfPG1* function during pathogenesis was evaluated by examining the disease phenotypes and gene expression patterns in *M*. *fructicola MfPG1*-overexpressing strains and in strains carrying the β-glucuronidase (GUS) reporter gene fused with *MfPG1* (*MfPG1-GUS*). The MFPG1-GUS reporter was expressed *in situ* in conidia and hyphae following inoculation of flower petals, and qRT-PCR analysis confirmed *MfPG1* expression during pathogenesis. *MfPG1*-overexpressing strains produced smaller lesions and higher levels of reactive oxygen species (ROS) on the petals of peach and rose flowers than the wild-type strain, suggesting that *MfPG1* affecting fungal virulence might be in part resulted from the increase of ROS in the *Prunus*–*M*. *fructicola* interactions.

## Introduction

Brown rot blossom blight and fruit rot caused by the fungal pathogen *Monilinia fructicola* (G. Wint.) Honey and related *Monilinia* species is a severe disease problem of stone fruits worldwide. The disease causes extensive tissue maceration in affected blossoms and fruits. Biochemical analyses suggest that cell wall-degrading enzymes (CWDEs) produced by the pathogen play a critical role during *M*. *fructicola* pathogenesis [1]. *M*. *fructicola* is able to produce and secrete pectin lyases and polygalacturonases in axenic cultures. The addition of pectic substances increases the production of CWDEs substantially, implicating an important role of CWDEs in nutrient acquisition. The exact function of CWDEs in *M*. *fructicola* pathogenesis is remains largely uncertain, although our previous studies have shown that the principal cutinase, MFCUT1, is a virulence factor [2].

Fungi produce a wide array of CWDEs and a number of these are critical for virulence in phytopathogenic fungi [3]. Endopolygalacturonases (endo-PG; EC 3.2.1.15), which cleave internal O-glycosidic bonds of pectate polymers in plant cell walls, are among the most important CWDEs in the plant–microbe interactions. Multiple endo-PG genes have been isolated and characterized in *Botrytis cinerea* and *Sclerotinia sclerotiorum* which, like *M*. *fructicola*, are members of the family Sclerotiniaceae. *B*. *cinerea* contains six differentially regulated endo-PG genes, including *BcPG1* and *BcPG2* that are expressed constitutively in culture, *BcPG4* and *BcPG*6 that are strongly induced by galacturonic acid, and *BcPG3* that is preferentially expressed at low pH [4]. Inactivation of the *BcPG1* gene in *B*. *cinerea* resulted in a strain that causes significantly smaller lesions on tomato leaves relative to the wild-type, supporting a virulence function of this endo-PG in gray mold disease [5]. In contrast, genetic analysis of *T4BcPG1*, a *BcPG1* homologue in the T4 strain of *B*. *cinerea*, revealed that this endo-PG can act as an elicitor to induce defense responses in grapevine [6]. *S*. *sclerotiorum* has four endo-PG coding genes [7]. Among them, only *SSPG1d* is highly expressed during infection [7]. These findings point to the complex role of fungal endo-PGs during pathogenesis and suggest that the expression of these CWDEs must be tightly coordinated for optimal colonization of the host by the pathogen.

Previously we have established a DNA transformation system for *M*. *fructicola* [8] and demonstrated that formation of appressorium and expression of *MfCUT1* (a cutinase gene) are required to penetrate into plant tissue [2,9]. To provide a better understanding of *M*. *fructicola* pathogenicity, we focused on genes that are involved in plant cell wall degradation and investigated their function on brown rot disease development. Since the genome sequence of *M*. *fructicola* is not yet available, we cloned five *M*. *fructicola* endoPG genes–*MfPG1*, *MfPG2*, *MfPG3*, *MfPG5* and *MfPG6* by PCR and determined their expression in axenic culture and *in planta* during pathogenesis. De Cal and colleagues have shown that *M*. *fructicola* could acidify the infected tissue during colonization on peach and nectarine fruit and that four of the *MfPGs* were regulated by pH in axenic culture [10]. However, the involvement of the five *MfPGs* in fungal growth and pathogenesis remains to be determined. The pathological role for *MfPG1* in lesion development was assessed by examining the host reaction after inoculation with wild-type and *MfPG1*-overexpressing strains of *M*. *fructicola*. Spatial and quantitative expression of *MfPG1* in inoculated host tissue was determined by *in situ* detection of β-glucuronidase (GUS) fused with MFPG1 and by qRT-PCR. The results indicate that overexpression of *MfPG1* actually increases reactive oxygen species (ROS) accumulation and reduces lesion development in *M*. *fructicola* host interactions.

## Results

### Cloning and characterization of *MfPG* genes

Five endopolygalacturonase (endo-PG) genes, designated *MfPG1*, *MfPG2*, *MfPG3*, *MfPG5* and *MfPG6*, were cloned from *M*. *fructicola* (S1 Table). Analysis and comparison of the assembled sequences from cDNA and genomic DNA revealed that *MfPG1* is an intronless gene, while the other four genes contain one to four introns with sizes from 49 to 62 bp. The MFPG1, MFPG2, MFPG5 and MFPG6 proteins have predicted molecular weights ranging from 35 to 37 kDa. In contrast, the MFPG3 protein has a predicted molecular weight of 50.1 kDa. MFPG1, MFPG2 and MFPG3 are likely basic PG proteins with a predicted pI of 9. MFPG5 and MFPG6 have a predicted pI of 5.

**Fig 1:**
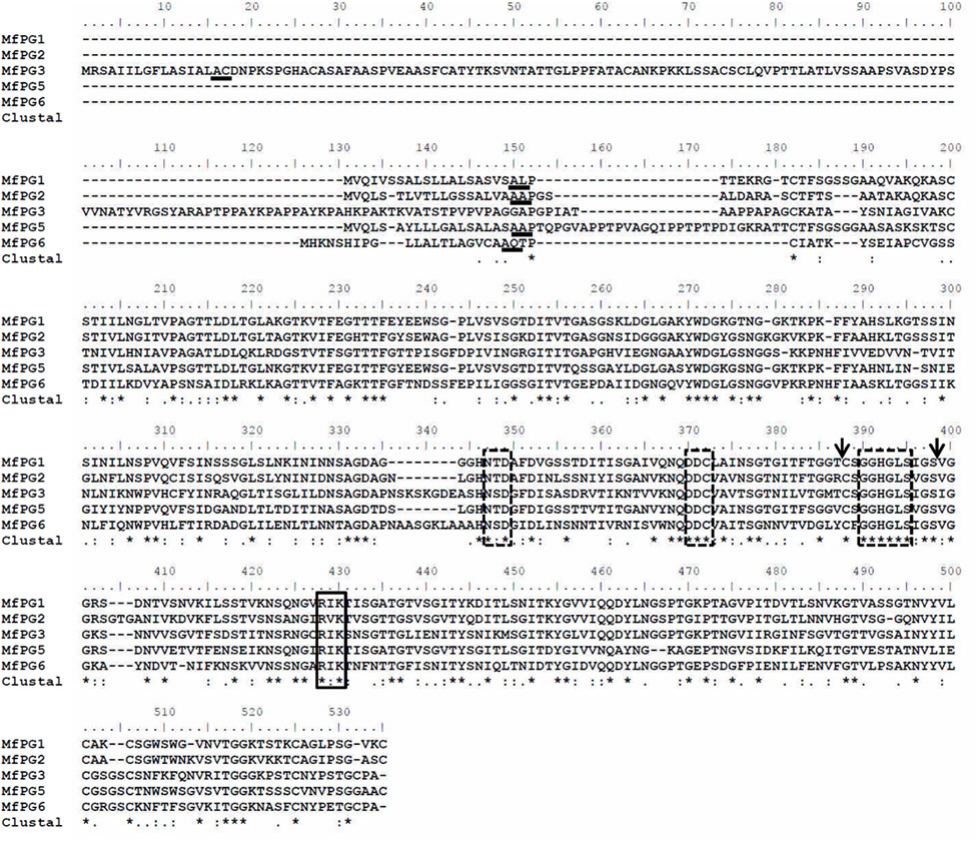
Amino acid alignment and functional domains of MFPG1, MFPG2, MFPG3, MFPG5 and MFPG6 of *M*. *fructicola*. Signal peptide cleavage sites (underline), polygalacturonase active site consensus (boxed with dash line) and polygalacturonase signatures (within two arrows), and substrate binding domains (boxed with solid line) are indicated. Consensus nucleotides are marked with stars.

The conceptually translated MFPGs belong to the glycosyl hydrolase family 28 which, after protein domain annotation, includes polygalacturonase and rhamnogalacturonase A [11]. All MFPGs contain sites for substrate hydrolysis (NT/SD, DDC and GGHGLS) and for substrate binding (RI/VK) (Fig 1). The consensus sequence (CSGGHGLSI/VGS) required for polygalacturonase activity was found at the C-terminus of all MFPGs. A secretory signal sequence of 16–21 residues was found at the N-terminus of all MFPGs.

Phylogenetic relationships of the *M*. *fructicola* MFPGs to other PG protein homologs from fungi, oomycetes and plants revealed that all PG proteins found in the family of Sclerotiniaceae can be grouped into six monophyletic clades (S1 Fig). Interestingly, PG3 and PG6 are less related to PG1, PG2, and PG5 than to the PG proteins from *Penicillium griseoroseum* (PgGII) and *Aspergillus niger* (PGAII)

Analysis of the 687-bp or 1-kb sequences upstream of the putative ATG translational start codon of each of the *MfPG1*, *MfPG2*, *MfPG3* and *MfPG5* genes identified several putative binding sites for diverse transcriptional regulators (S2 Fig). All *MfPG* promoter regions contain at least one carbon catabolite repressor (CreA) binding site and the nitrogen-inducible activator (AreA/NIT2) sites. A pH-responsive transcription factor pacC binding site was found 876 and 90 nucleotides upstream from the ATG codon of *MfPG*3 and *MfPG5*, respectively. No pacC binding sites was found in the promoter of *MfPG1* and *MfPG2*. The promoter region of all *MfPGs* except *MfPG3* contain multiple TATA boxes (TBP; -76 and -26), a binding site for the activator of RNA polymerases. The *MfPG6* upstream region was not available for this analysis since only 60-bp sequence was obtained.

### Differential expression of *MfPGs* in *M*. *fructicola* grown in axenic cultures

The expression of polygalacturonase gene family members has been known to be regulated by pH in many fungal pathogens [12]. Northern blot hybridization revealed that the *MfPG1* gene was preferentially expressed under acidic conditions. However, the expression of *MfPG2*, *MfPG3*, *MfPG5* and *MfPG6* could not be detected by Northern blotting. The expression level of each *MfPG* gene was further analyzed by qRT-PCR (S3 Fig). *MfPG1* expression was dominant at pH 3.5 to 4.0. *MfPG2* expressed preferentially at pH values 3.5 and 6.0. The expression of *MfPG3* and *MfPG6* was favored when the fungus was grown at neutral pH, while the expression of *MfPG5* was slightly higher when the fungus was grown at pH 4.0 than at the other pH’s tested. Comparison of the expression levels among five *MfPGs* confirmed further that *MfPG1* was the predominant homolog expressed *in vitro* (S2 Table). At the end of the growth period, the pH in all media did not change significantly.

**Fig 2:**
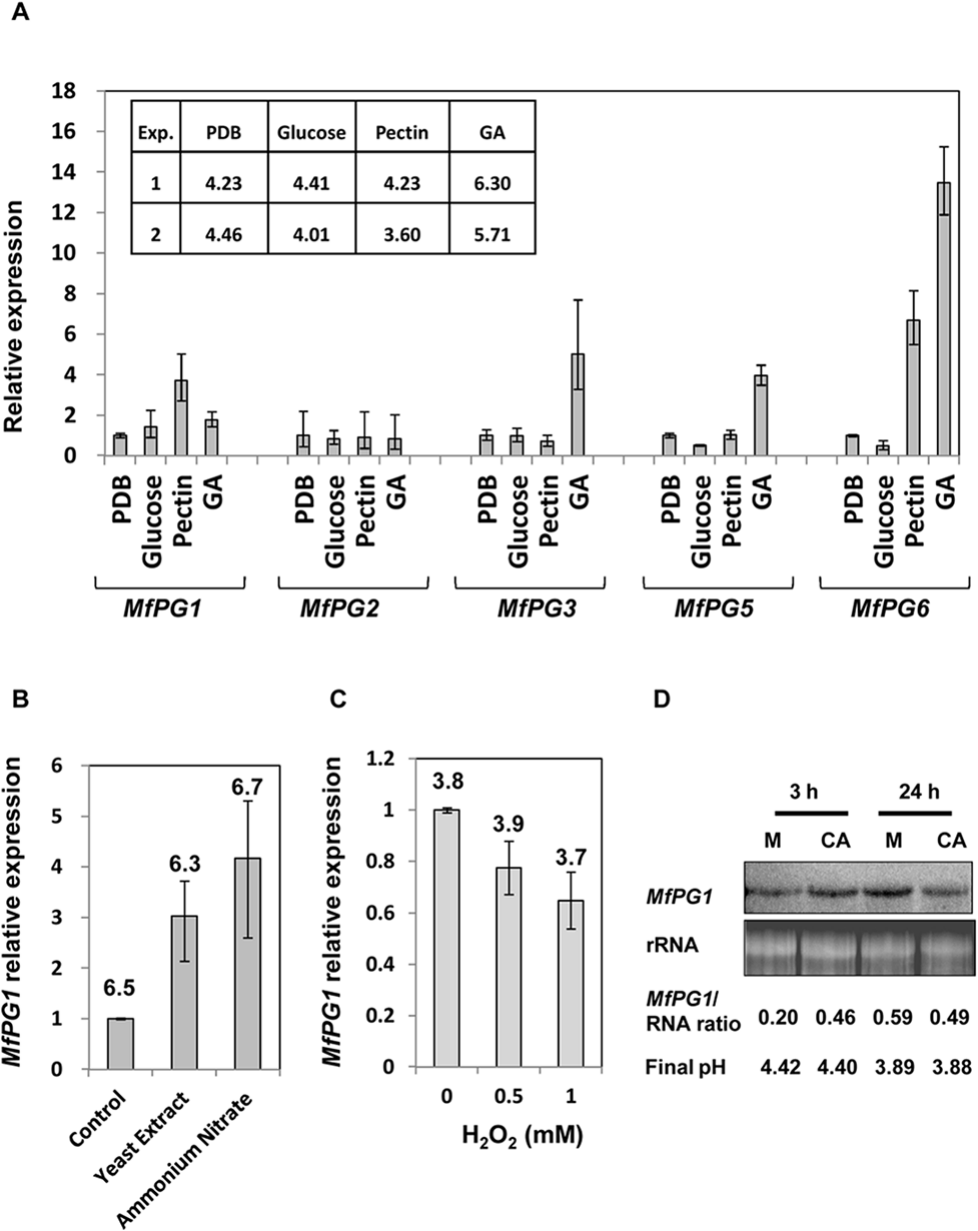
Expression of *MfPGs* (A) or *MfPG1* (B, C and D) in *M*. *fructicola* cultured in modified Czapek-Dox salts supplemented with different sole sources of carbon (A), yeast extract or ammonium nitrate as the sole nitrogen source (B) or in the presence of H_2_O_2_ (C) or caffeic acid (D). Gene transcripts were detected by qRT-PCR (A, B and C) or by Northern blot (D). The expression of *MfPGs* or *MfPG1* was compared to that of the PDB treatment (A), control (B) and 0 mM H_**2**_O_**2**_ (C). The final pH of each treatment is also indicated. GA, galacturonic acid.

Expression of the polygalacturonase genes has been known to be regulated by carbon and nitrogen sources [13]. Because CreA binding sites were found in the 5’ untranslated region of *MfPGs*, the expression of *MfPGs* was examined in *M*. *fructicola* grown in medium amended with various carbon sources. Expression of the *MfPG1* gene was higher when the fungus was grown in pectin-containing medium than in PDB or medium containing galacturonic acid or glucose as the sole carbon source (Fig 2A). The *MfPG3*, *MfPG5* and *MfPG6* genes were expressed to high levels when the fungus was grown in medium amended with galacturonic acid. Expression of the *MfPG6* also was enhanced by pectin. The *MfPG2* gene was expressed at low levels regardless of carbon sources. There was no significant difference in the accumulation of the *MfPG2* gene transcript when the fungus was grown in medium containing various carbon sources. Glucose apparently repressed the expression of *MfPG5*.

Because AreA/NIT2 binding sites were found in the 5’-untranslated region of *MfPGs*, expression of *MfPGs* was also examined in *M*. *fructicola* grown in medium amended with different nitrogen sources (Fig 2B). Only expression of the *MfPG1* gene was greatly enhanced when the fungus was grown in medium containing ammonium nitrate or yeast extract as the sole nitrogen source.

Our previous studies have shown that H_2_O_2_ and caffeic acid impact gene expression and virulence of *M*. *fructicola* [14]. The expression of *MfPG1* did not change after *M*. *fructicola* was shifted to a H_2_O_2_-containing medium. However, qRT-PCR analysis revealed that expression of *MfPG1* was slightly inhibited when H_2_O_2_ was added directly to *M*. *fructicola* cultures (Fig 2C). As assessed by Northern blotting, *MfPG1* transcript levels increased when *M*. *fructicola* was shifted to medium containing caffeic acid for 3 h and slightly decreased after 24 h (Fig 2D). The pH changes in culture filtrates among the treatments were within 0.2 units.

### *MfPGs* are differentially expressed during *M*. *fructicola* pathogenesis

**Fig 3:**
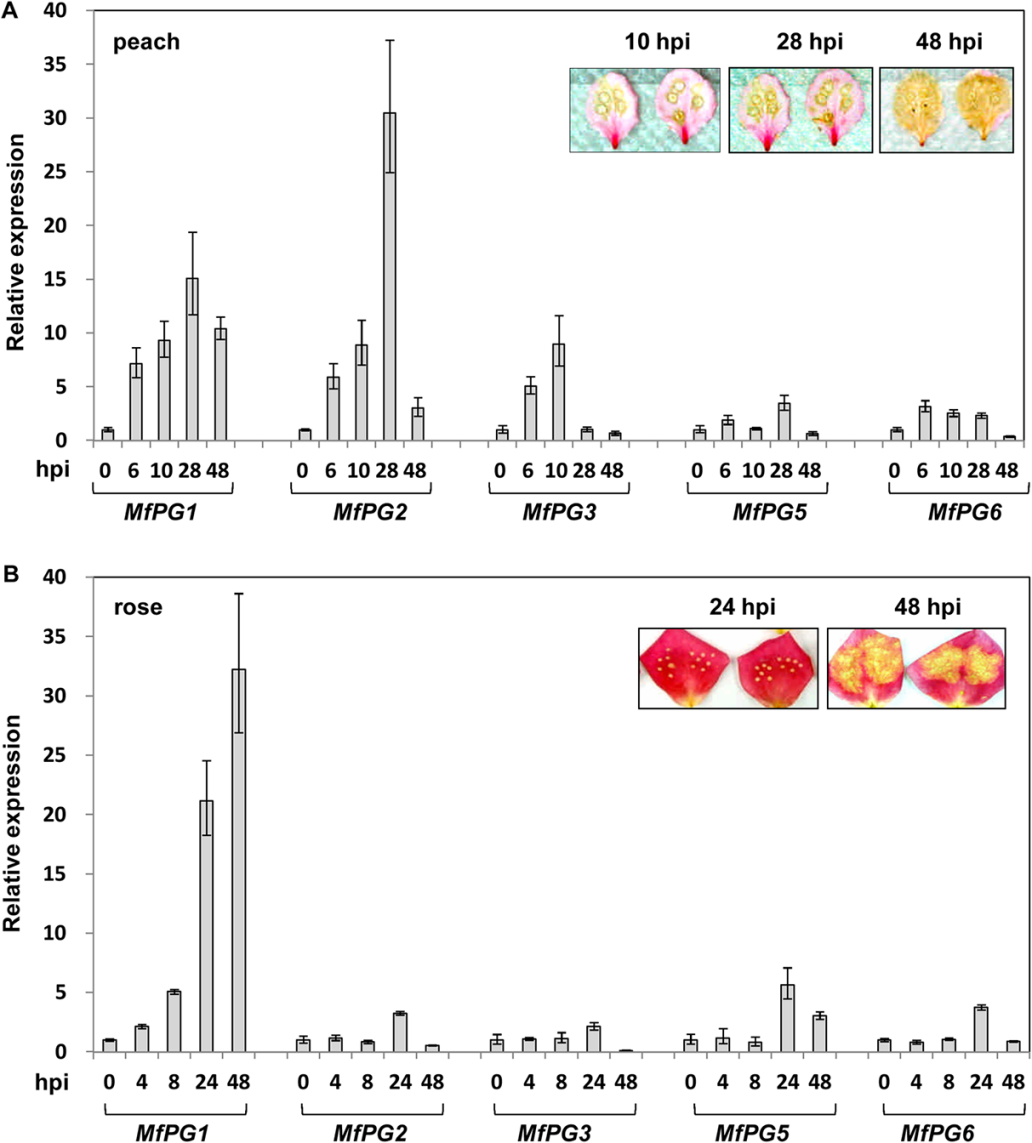
Quantitative reverse transcriptase PCR analysis of *MfPGs* expression in *M*. *fructicola* inoculated onto peach (A) and rose (B) petals hour post-inoculations (hpi). The expression levels of the *MfPGs* genes at different time points were compared to 0 hpi using the comparative C_**T**_ method. Brown rot lesion development is also presented (inset).

All *MfPG* gene transcripts could be detected to various levels in *M*. *fructicola* inoculated onto peach petals at 6 h post inoculation (hpi) (Fig 3A). Accumulation of the *MfPG1*, *MfPG2* and *MfPG3* gene transcripts increased significantly during symptom development. Although the *MfPG5* and *MfPG6* transcript levels did not change dramatically, their expression was up-regulated in *M*. *fructicola* during pathogenesis in peach petals. On rose petals inoculated with *M*. *fructicola*, *MfPG1* transcript levels were abundant and increased over time during colonization (Fig 3B). The expression level of all five *MfPG* genes was compared, revealing that *MfPG1* was expressed at the highest level at each time point on peach and rose petals (S3 Table). Overall, *MfPG1* gene was expressed equally well both in culture and *in planta* (S2 and S3 Tables). Both *MfPG2* and *MfPG3* were highly expressed *in planta* compared to their expression in axenic cultures. The expression of *MfPG5* and *MfPG6* was lower *in planta* than in axenic cultures.

### Expression of the *MfPG1*-GUS reporter during pathogenesis

**Fig 4:**
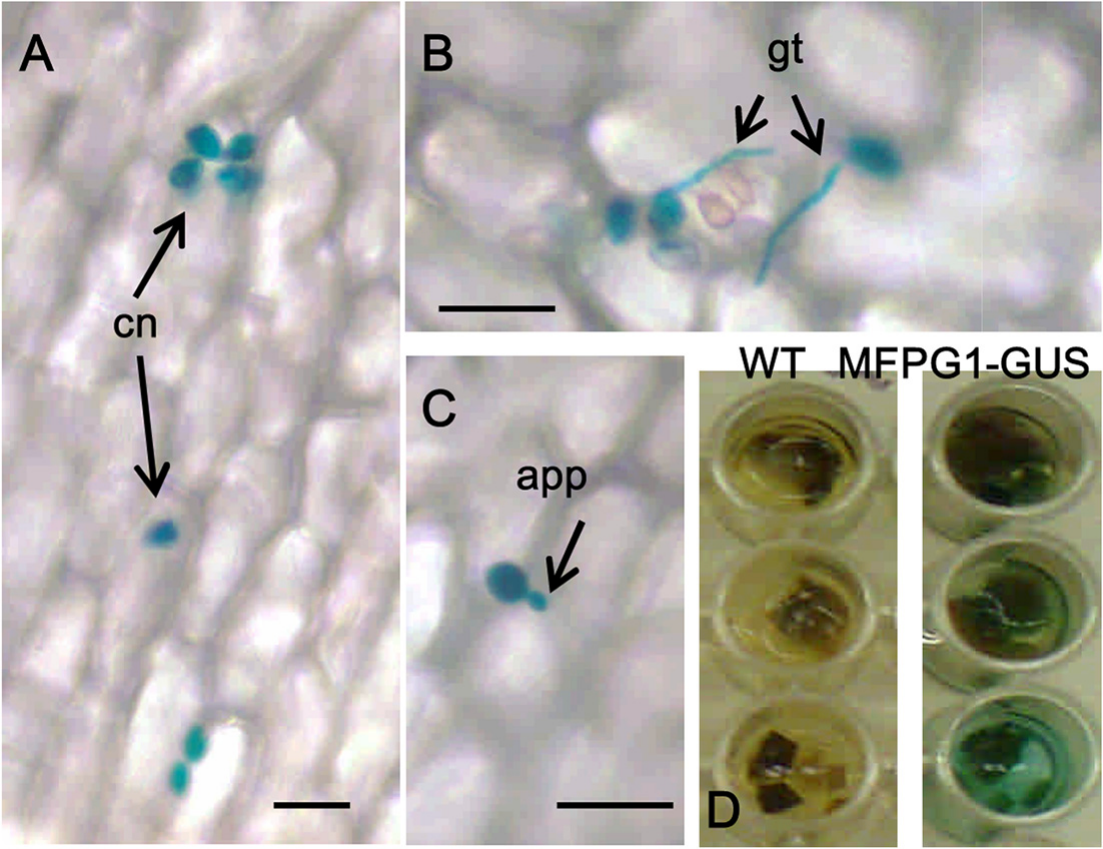
Expression of the MFPG1-GUS fusion protein in *M*. *fructicola*. Fungal strain expressing the pMfPG1-GUS construct was inoculated onto on peach petals (A, B, C) and apple tissues (D). GUS activity was detected in non-germinated conidia (A), germinated conidia (B), appressoria (C) and infected apple tissues (D), after stained with X-gluc. No GUS activity was observed in the wild-type strain (WT). app, appressorium; cn, conidium; gt, germ tube. Bar = 100 μm.

An MFPG1-GUS fusion protein was constructed to examine the expression pattern of *MfPG1* during *M*. *fructicola* pathogenesis. Transformation of pNC-MFPG-GUS plasmid into fungal protoplasts recovered five transformants (PG20, PG50, PG55, PG213, and PG249) showing blue pigments on pectin-containing medium after staining with X-gluc, indicative of GUS activity. The colonies of the wild-type strain were not stained blue under similar culture conditions. GUS activity was detected in these transformants inoculated on peach petals at 5 hpi (Fig 4). Expression of MFPG1-GUS was also detected in transformants on apple and peach fruits at 4 dpi. Microscopic examination revealed MFPG1-GUS expression in conidia, germ tubes and appressoria during penetration on peach petals. GUS activity was not detectable in wild-type inoculated on peach petals or apple fruit.

### Fungal strains overexpressing *MfPG1* induce smaller brown rot lesions and higher ROS levels

**Fig 5:**
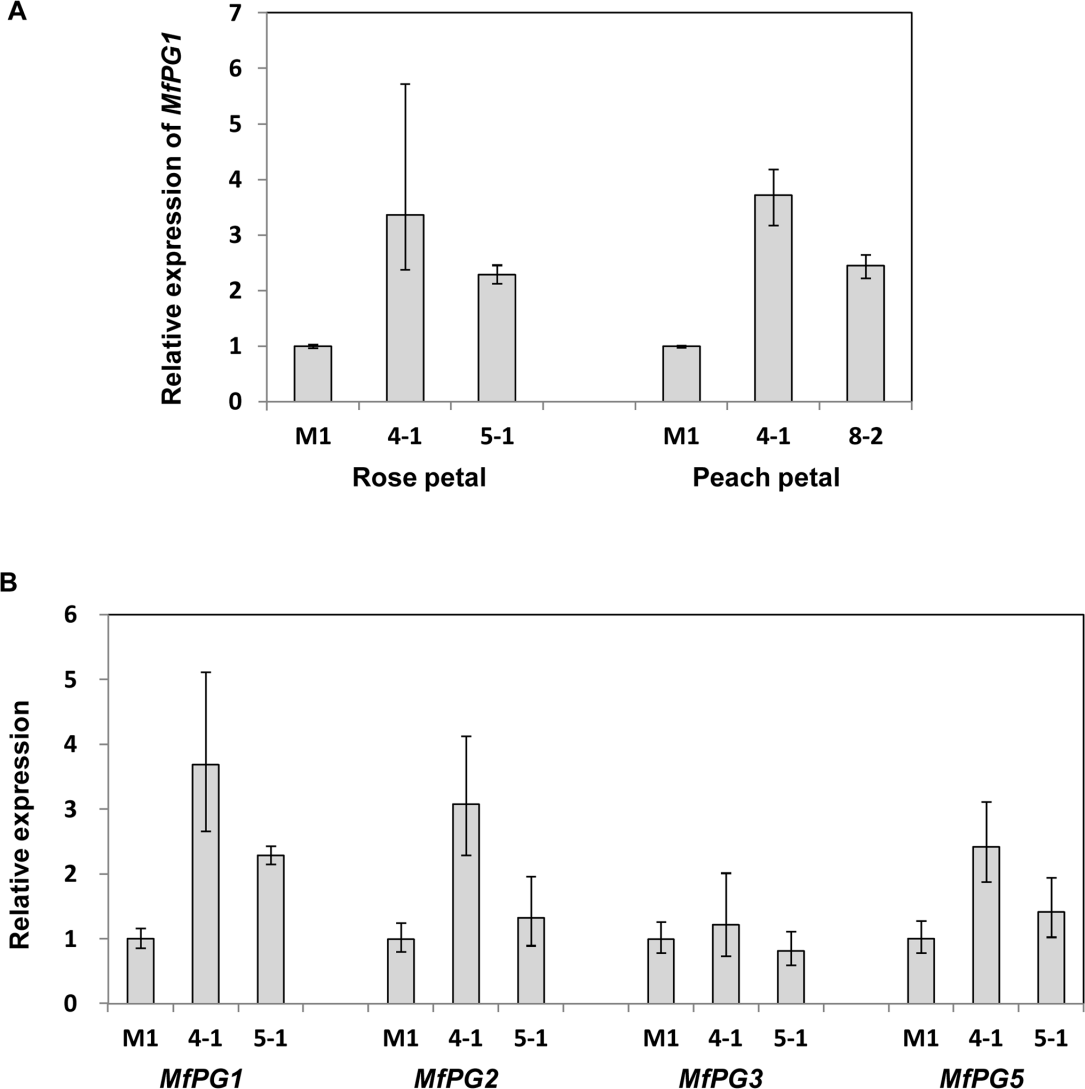
Expression of *MfPG1* in *M*. *fructicola* strains inoculated onto host plants. (A) Quantitative reverse transcriptase PCR analysis of *MfPG1* expression in the wild-type (M1) strain and three *MfPG1*-overexpressing strains (4–1, 5–1 and 8–2) of *M*. *fructicola* inoculated on rose and peach petals at 24 hpi. (B) Expression of *MfPG1*, *MfPG2*, *MfPG3* and *MfPG5* in *M*. *fructicola* strains inoculated on rose petals at 24 hpi. The ΔCt values representing the expression levels of *MfPGs* in different mutant strains were compared to that of wild-type using the comparative C_**T**_ method.

Southern blot hybridization of genomic DNA cleaved with various restriction endonucleases to a *MfPG1* gene probe revealed that *M*. *fructicola* contains a single copy of *MfPG1* (S4 Fig). The *MfPG1* gene was over-expressed in *M*. *fructicola* to assess how this would influence virulence. Transformation of a pUCATH-MFPG1 plasmid into *M*. *fructicola* recovered seven transformants carrying multiple *MfPG1* genes (S5 Fig). The *MfPG1*-overexpressing strains (4–1, 5–1, and 8–2) produced significantly smaller brown rot lesions than those induced by the wild-type strain on rose and peach petals (Table 1). qRT-PCR analysis revealed that *MfPG1* mRNA levels were significantly higher in both rose and peach petals inoculated with the 4–1, 5–1 and 8–2 strains than in host tissues infected with the wild-type strain M1 (Fig 5A). Expression of the *MfPG2*, *MfPG3*, *MfPG5* and *MfPG6* genes was up-regulated at 24 hpi in rose petals inoculated with the *MfPG1*-overexpressing strain (Fig 5B and S4 Table). The accumulation of transcripts for *MfPG2* and *MfPG5* was significantly higher in the overexpression strains (4–1 and 5–1) than wild-type inoculated on rose and peach petals. The pH of petal tissue changed during the course of pathogenesis, decreasing from pH 5.5 at 0 hpi to 4.5 at 48 hpi. Assays for enzyme activity revealed that the *MfPG1*-overexpressing strains displayed higher polygalacturonase activity than the wild-type strain (S6 Fig). No notable differences in growth, germination, and appressorium formation were observed between the wild-type strain and the overexpression strains in axenic culture and *in planta*.

**Table 1:** Pathogenicity of *MfPG1*-overexpressing strains (4–1, 5–1 and 8–2) assayed on rose petals and peach petals 18 to 30 h post-inoculation.

Plant	Exp.	N^a^	Strain^b^	Mean lesion area (mm^2^)	*p*-value^c^
Rose petal	1	16	WT	23.28	<0.05
			4–1	12.91	
Rose petal	1	19	WT	21.19	<0.05
			5–1	16.94	
Rose petal	1	18	WT	18.31	0.09
			8–2	8.00	
Rose petal	2	16	WT	13.96	<0.05
			8–2	9.22	
Peach petal	1	29	WT	19.66	0.06
			4–1	16.57	
Peach petal	1	26	WT	18.16	<0.05
			5–1	13.84	

^a^ N, numbers of plant sample tested.

^b^ WT, wild type

^c^ Statistical analysis was performed by a pair *t*-test.

**Fig 6:**
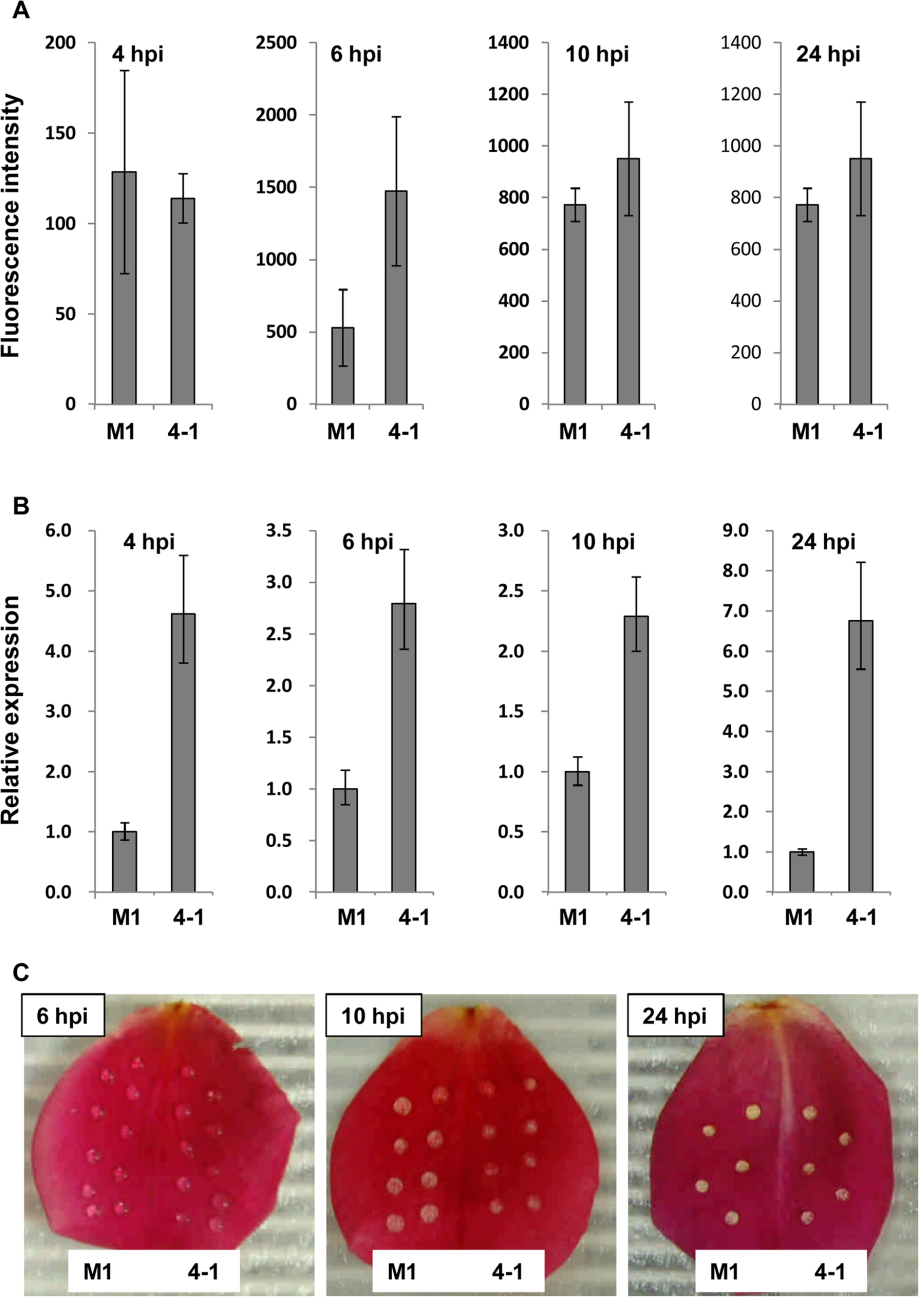
Reactive oxygen species accumulation (A), *MfPG1* expression (B) and lesion development (C) on rose petals inoculated with the wild-type (M1) strain and *MfPG1*-overexpressing strain 4–1. (A) Reactive oxygen species was detected with the oxidatively active fluorescent dye 2,7-dichlorofluorescin diacetate (DCFH-DA) at 4, 6, 10 and 24 hpi. (B) Expression of *MfPG1* in *M*. *fructicola* strains was detected by qRT-PCR. The expression level of *MfPG1* in the overexpressing strain was compared to that of wild-type using the comparative C_**T**_ method. (C) The wild-type (M1) and overexpressing (4–1) strains were paired inoculated on rose petals and lesion developments at 6, 10 and 24 hpi were presented.

Reactive oxygen species (ROS) are associated with cell death and often with the development of hypersensitive resistance. ROS accumulation was measured in rose petals after inoculation with the *MfPG1* overexpressors and the wild-type. *MfPG1*-overexpressing strains induced ROS accumulation at levels higher than the wild-type around the infection site at 6 hpi (Fig 6A and S7 Fig). The relative expression level of *MfPG1* in the *MfPG1-*overespressing strain was analyzed by qRT-PCR. The results revealed that the *MfPG1-*overexpressing strain expressed higher level of *MfPG1* than those of the wild-type strain at all examined time points (Fig 6B). Rose petals inoculated with the wild-type strain induced chlorotic lesions at the site of infection 10 hpi. Rose petals challenged with the *MfPG1-*overexpressing strain developed lesions at rates much slower than the wild-type strain (Fig 6C).

## Discussion

*M*. *fructicola* is a devastating pathogen on Rosaceae, causing blossom blight and fruit rot. Little is known about pathogenic mechanisms in *M*. *fructicola* and related *Monilinia* species. In the present study, five endo-PG genes from *M*. *fructicola* were cloned and characterized and functional analysis suggested that *MfPG1* impacts fungal virulence, as the *MfPG1*-overexpressing strains induced smaller brown rot lesions than the wild-type strain on rose and peach petals. The five PG genes were found to be differentially expressed in culture and in inoculated host tissue. These different patterns of expression may reflect a degree of selectivity in substrate utilization among the various homologs during saprophytic and parasitic growth.

All five *MfPG* genes from *M*. *fructicola* share sequence similarities of endo-PG genes from *B*. *cinerea* and *S*. *sclerotiorum*, both members of the Sclerotiniaceae as is *M*. *fructicola*. However, the endo-PGs found in *B*. *cinerea* and *S*. *sclerotiorum* share greater similarity with each other than with their counterparts in *M*. *fructicola*. This suggests that *B*. *cinerea* and *S*. *sclerotiorum* are evolutionarily closer than *M*. *fructicola*, and analysis of rDNA sequences corroborates this genetic correspondence among these species [15].

The production of plant CWDEs, often regulated by transcription factors, such as CreA, AreA/NIT2 and pacC, is important for fungal growth and pathogenicity *in vitro* and *in planta* [12,13,16,17,18,19,20]. Analysis of the upstream regions of *MfPG1*, *MfPG2*, *MfPG3* and *MfPG5* revealed a number of potential regulatory motifs that may contribute to the differential expression of these genes in response to experimental treatments. The promoter regions of *MfPG1*, *MfPG2* and *MfPG3* contain one or two CRE binding sites, but the promoter region of *MfPG5* has four CRE binding sites. qRT-PCR analysis revealed that expression of *MfPG5*, but not *MfPG1*, *MfPG2* and *MfPG3*, was down-regulated in medium containing glucose as the sole carbon source, providing strong evidence for carbon catabolite repression. When grown on pectin amended medium, *MfPG1*, *MfPG2* and *MfPG5* are preferentially expressed under acidic conditions, while *MfPG3* and *MfPG6* are expressed to high levels at neutral pH. The pacC binding site found in the promoter region of *MfPG3* might contribute to the up-regulation of this gene at neutral to alkaline condition. Although the promoter region of *MfPG5* also contains a putative pacC binding site, immediately adjacent to a TBP binding site, the expression of *MfPG5* was repressed at alkaline environment. pacC is a transcription factor that up-regulates alkaline-expressed genes and downregulates acid-expressed genes [16]. The expression patterns of all *MfPG* genes in response to pH are consistent with the reported findings [10]. The pH regulation on the expression of *MfPG* genes might in part contribute to their expression during pathogenesis. For example, *MfPG1*, *MfPG2* and *MfPG5* were acid-inducible genes and up-expressed at the late stage of infection, in which the pH of infected petal tissue became acidic during pathogenesis. *MfPG1*, *MfPG3*, *MfPG5* and *MfPG6* were up-regulated in the presence of galacturonic acid and *MfPG5* transcripts were accumulated at higher level in *MfPG1*-overexpressing strain than the wild-type strain during infection. This indicates that *MfPG5* expression could be up-regulated by galcturonate released by MFPG1 during fungal infection. Galacturonic acid is a monosaccharide that is released from pectin after PG hydrolysis and has been shown to up-regulate the inducible gene family members in *S*. *sclerotiorum* [21]. Because the expression of *MfPG1*, *MfPG3*, *MfPG*5 and *MfPG6* is up-regulated by galacturonic acid and/or pectin in axenic culture, it is tempting to speculate that these MFPGs are functioning in cell wall degradation and nutrient acquisition during *M*. *fructicola* colonization within plant hosts.

Nitrogen has long been known to influence the production of pectolytic enzymes in fungi [13]. For example, ammonium enhanced polygalacturonase production by *Penicillium expansum* [22]. The transcription factors AreA in *Aspergillus nidulans* and NIT2 in *Neurospora crassa* are important regulatory proteins for nitrogen metabolism [13]. Four putative AreA/NIT2 binding sites are found in the *MfPG1* promoter region and one or two AreA/NIT2 binding sites are found in the promoter regions of *MfPG2*, *MfPG3* and *MfPG5*. However, only the expression of *MfPG1* is up-regulated when ammonium nitrate or yeast extract is used as the sole nitrogen source, suggesting that multiple AreA/NIT2 binding sites contribute to the unique regulation of *MfPG1* in response to different nitrogen sources both in axenic culture and *in planta*.

All five *MfPG* genes are up-regulated to different degrees in *M*. *fructicola* during host infection, suggesting a role during *M*. *fructicola* pathogenesis. The high level of *MfPG1* expression relative to other *MfGP* genes in axenic culture is similar to that observed during infection and colonization of host tissue. An interesting finding of this study is that *MfPG1* seems to play a negative role in fungal virulence. *MfPG1* is constitutively expressed in hyphae, conidia, appressoria and germ tubes during fungal colonization, as evidenced by MFPG1-GUS fusion protein expression. Although *M*. *fructicola* can be genetically transformed, generation of disruptants with homogeneous nuclei has proven to be difficult [2,8]. Similarly, we were unable to recover any homogeneous *MfPG1* mutants in this study after numerous attempts using a split-marker gene replacement approach [23]. To evaluate the role of *MfPG1* in pathogenicity, fungal strains overexpressing *MfPG1* were created and analyzed. The *MfPG1*-overexpressing strains accumulated higher levels of the *MfPG1* transcript than wild-type when they were inoculated onto rose and peach petals. The *MfPG1*-overexpressing strains induced smaller lesions than those induced by the wild-type, supporting that *MfPG1* suppresses virulence in these interactions. The smaller lesions observed in rose and peach petals inoculated with the *MfPG1*-overexpressing strains relative to the wild-type could be in part attributable to higher levels of ROS accumulation induced in the host tissue. The results suggest that the product of *MfPG1* could function as an elicitor, inducing plant defense, which partially limits lesion development. As demonstrated in the T4 strain of *B*. *cinerea*, a *BcPG1* homologue indeed elicits defense responses in grapevine [6]. In other phytopathogenic fungi, endo-PGs can function as virulence factors [5,6,24,25]. *MfPG1* could be required for generating the initial pectic fragments (oligogalacturonides) that in turn induce the expression of other *MfPG* genes as well. A recent study [26] also found that expression of *S*. *sclerotiorum SsSPG1* is significantly higher in plant tissues infected by a superoxide dismutase mutant than the wild-type strain. The superoxide dismutase mutant is less virulent and induces higher ROS accumulation than the wild-type strain on pea leaves.

Our previous work has demonstrated that the expression of *MfCUT1*, a virulence factor that encodes the principle cutinase is regulated by redox status in *M*. *fructicola* [2,14,27]. The expression of *MfCUT1* is induced by H_2_O_2_ and repressed by the fruit phenolic caffeic acid, a pattern that is opposite to what we observed with *MfPG1*. In contrast, expression of *MfPG1* is down-regulated by H_2_O_2_ and up-regulated by caffeic acid, suggesting a relation between the redox regulation and pathogenicity roles of *MfPG1* and *MfCUT1* during pathogenesis. Because overexpression of *MfPG1* in strains results in smaller lesions induced by those strains, the display of *MfPG1* must be correctly orchestrated during infection for optimal virulence.

In spite of their worldwide impact as devastating pathogens of orchard crops, there have been relatively few studies of pathogenic mechanisms in *M*. *fructicola* and related *Monilinia* species. However, recent advances in experimental methods and the genomic tools coming on-line are enabling mechanistic studies that will lead to a deeper understanding of pathogenesis by *Monilinia* species.

## Materials and Methods

### Strains and cultivation

The wild-type MUK-1 (M1) strain of *M*. *fructicola* used in this study was single-spore isolated from diseased peach fruit and has been previously characterized [28]. Fungal strains were cultured at 23°C. Plasmids were propagated in *Escherichia coli* XL1-Blue competent cells (Stratagene, La Jolla, CA).

### Cloning and sequencing of *MfPGs* and their flanking regions

Oligonucleotide primers used in this study are given in S5 Table. A 1.0-kb *MfPG1* DNA fragment was amplified from genomic DNA of *M*. *fructicola* with the primers MFPG-F2 and MFPG-R1 that are complementary to highly conserved regions of the endo-PG-coding genes from *B*. *cinerea* (*BcPG1*; Accession No. U68715) and *S*. *sclerotiorum* (*SsPG1d*; Accession No. L29041). A 0.7-kb *MfPG2* DNA fragment was amplified with the primers MFPG2-F2 and MFPG2-R1.

Additional endo-PG-coding genes were amplified by PCR with degenerate primers using a TaKaRa EX Taq DNA polymerase (TAKARA Bio INC., Japan). Two DNA fragments of sizes 320 and 400 bp were amplified from *M*. *fructicola* genomic DNA with the primers MfPGDP-F2 and MfPGDP-R2. The primers MFPG6-DF1 and MFPG6-DR1, which are complementary to a PG6 homolog of *B*. *cinerea* (Accession no. AAM22178) amplified a 1.0-kb DNA fragment. PCR amplicons were cloned into a pGEM-T easy vector (Promega) for sequence analysis. DNA fragments amplified with the primers MfPG3-F1/MfPG3-R1, MfPG5-F1/MfPG5-R1 or MfPG6-F1/MfPG6-R1 were directly sequenced for further confirmation of sequence identity. The 5’- and 3’-flanking regions of each of the *MfPG* genes were amplified by PCR with inverse primers from *Bam*HI-, *Eco*RI-, *Sac*I-, *Xba*I-, *Bgl*II- or *Hind*III-digested and self-ligated genomic DNA templates from *M*. *fructicola* using a High fidelity KOD HiFi polymerase kit (Novagen, Madison, WI) as described previously [2]. *MfPG* cDNA was amplified independently from total RNA of fungal mycelium (*MfPG1*) or of *M*. *fructicola*-infected rose petals (*MfPG2*, *MfPG5* and *MfPG6*) with gene-specific primers and sequenced. Open reading frame (ORF) and exon/intron positions were identified by comparison of genomic and cDNA sequences. Sequence data were deposited within the EMBL/GenBank Data Libraries under accession nos. KC597703 (*MfPG1*), KC597704 (*MfPG2*), KC597705 (*MfPG3*), KC597706 (*MfPG5*) and KC597707 (*MfPG6*).

### MFPG1-GUS fusion and *MfPG1* overexpression constructs and fungal transformation

The MFPG1-GUS fusion construct was made in the binary vector pNC1381Xa carrying an *nptII* cassette and *gusA* ORF [2]. A 2.4-kb fragment encompassing the entire *MfPG1* ORF and its 5’-untranslated region was amplified from genomic DNA with the primers MFPG-EcoRI-901 and MFPG-SpeI-3026. After digestion with *Eco*RI and *Spe*I, the amplicon was cloned into pNC1381Xa to produce pNC-MFPG-GUS. For *MfPG1* overexpression, a DNA fragment containing the entire *MfPG1* ORF and its 5’- (1 kb) and 3’-untranslated (0.3 kb) regions was amplified from genomic DNA with the primers MFPG1-SmaI and MFPG1-SacI using the KOD HiFi polymerase kit. The amplicon was digested with *Sma*I and *Sac*I and then cloned into pUCATPH [29] to generate the plasmid pUCATPH-MFPG1. The resultant plasmids were transformed into protoplasts prepared from the wild-type strain of *M*. *fructicola* as previously described [2]. For transformation, 10 μg DNA was mixed with 10 μl spermidine (50 mM) and 200 μl protoplast (5 x 10^7^/ml). After 30 min incubation in ice, 1.25 ml PEG solution (40% PEG, 50 mM CaCl_2_, 50 mM Tris-HCl, pH 8.0) was added into the mixture and incubated at room temperature for an additional 25 min. The protoplasts were spread on SH medium [30] supplemented with G418 sulfate or hygromycin.

### Expression of *MfPG*s

To examine the influence of pH, nitrogen source and exogenous redox compounds on the expression of *MfPGs*, freshly prepared conidial suspensions (2×10^5^/ml) were transferred to a 9-cm petri dish with 20-mL starter medium containing 0.1% yeast extract, 0.1% glucose, and modified Czapek-Dox salts [14]. After incubating for 3 days, fungal mycelium was washed twice and then immersed in 20 mL modified Czapek-Dox salts supplemented with 1% citrus pectin (second medium). The pH of second medium was adjusted to appropriate values with a 0.1 M sodium citrate/phosphate buffer (pH 3.5, 4.0, 5.0, 6.0 and 7.0) or 0.1 M phosphate buffer (pH 8.0). To determine the effect of nitrogen sources, the second medium (pH 6.0) was supplemented with 0.1% yeast extract or 0.1% ammonium nitrate. To determine the effect of caffeic acid, the second medium was supplemented with 1% pectin as previously described [14]. Mycelia were harvested at 3 or 24 h post-incubation (hpi) for RNA isolation.

To assess the effect of different carbon sources on the expression of *MfPGs*, fungal strain was grown in 20 ml PDB medium (pH 6.0) or medium containing modified Czapek-Dox salts (pH 6.0), 0.1% yeast extract and a carbon source (1% glucose, 1% pectin or 1% galacturonic acid) for 48 h. To verify the effect of H_2_O_2_ on *MfPG1* expression, fungal spores were inoculated into a 20 ml medium containing modified Czapek-Dox salts (pH 6.0), 0.1% yeast extract and 1% pectin for 48 h cultivation and then treated with H_2_O_2_. Each treatment had three replicates within an experiment and at least two independent experiments were performed.

### Extraction and manipulation of nucleic acids

Fungal DNA and RNA extraction was conducted as described (Lee *et al*., 2010). RNA was extracted from inoculated plant tissues using a RNA extraction kit (GeneMark, Taiwan) or from mycelia as described previously [14]. Standard procedures were used for endonuclease digestion of DNA, electrophoresis, and Southern- and Northern-blot hybridizations. DNA probes used for hybridization were labeled with digoxigenin (DIG)-11-dUTP (Roche Applied Science) by PCR with gene-specific primers. Probe labeling, hybridization (at 55°C), post-hybridization washing and immunological detection of the probe using a CSPD chemiluminescent substrate for alkaline phosphatase were conducted according to the manufacturer’s recommendations (Roche Applied Science).

### Real-Time quantitative reverse transcriptase PCR

Purified RNA was treated with DNase and used to synthesize cDNA with the MMLV high performance reverse transcriptase (Epicentre, USA) and an oligo-dT primer (5’-T25VN-3’). Primers used for quantitative PCR (qPCR) were designed using the Roche on-line design tool (http://www.roche-applied-science.com/sis/rtpcr/upl/index.jsp?id=UP030000) (S5 Table). The qPCR was conducted with a Rotor-Gene Q PCR machine (QIAGEN) using HOT FIREPol EvaGreen qPCR Mix Plus kit (Solis BioDyne). The cycling profile for amplification was the following: 95°C for 15 min, followed by 45 cycles of 95°C for 20 s, 58°C for 15 s and 72°C for 15 min. Amplification of specific transcripts was confirmed by melting curve and agarose gel electrophoresis staining with ethidium bromide. Amplification of the *M*. *fructicola* β-tubulin gene was used as an internal control. The ΔCt values (MfPG Ct– β-tubulin Ct) from three replicates in each experiment were used to calculate the relative expression levels using a comparative C_T_ method [31].

### Plant inoculations

Assays for fungal pathogenicity were performed on detached flower petals of peach (cv. “TianTao”) and rose (Rosa “Flaming Peace”) as well as apple fruits (*Malus domestica* cv. “Fuji”). The hybrid tea rose cv. Flaming Peace was identified as an alternative host to complement pathogenicity assays of *M*. *fructicola* on stone fruits, which are seasonally restricted [14]. Apple fruits were wounded prior to inoculation. Inoculation was performed by applying droplets (5 μl) of conidial suspensions (10^5^ spores/ml) onto host surface. Lesion size was measured as previously described [2] and analyzed statistically with the Excel paired *t*-test.

### Detection of ROS

Conidia suspension (1x10^5^/ml) prepared from the wild-type strain and *MfPG1*-overexpressing strains were inoculated onto rose petals (30 μl per drop). Reactive oxygen species (ROS) was detected by 2,7-dichlorofluorescin diacetate (DCFH-DA) applying on the inoculation site at a final concentration of 60 μM. The treated petals were incubated at 37°C for 30 min and examine for fluorescence. The fluorescence was measured with a multimode reader (Infinite M200, Tecan Group) with excitation wavelength at 485 nm and emission wavelength at 535 nm.

### Enzyme activity assays

Fungal strains were cultured in modified Czapek-Dox salts supplemented with 0.1% yeast extract and 1% polygalacturonate for six days and culture filtrate was collected for enzymatic activity detection using a cup plate assay. The culture filtrate was added into a 3-mm-diameter well of agar plate containing polygalacturonate (0.5%), agar (2%) and sodium acetate (0.1 M, pH 5.3) and then incubated for 8 h at 37°C. Area of clear zone, indicative of polygalacturonase activity, was measured after the plate was flooded with 1% cetyl trimethyl ammonium bromide [32].

### Bioinformatics

Database searches and comparisons were performed using the BLAST network service available at the National Center of Biotechnology Information (http://www.ncbi.nlm.nih.gov/). The cleavage site to remove the secretary signal peptide was identified using the SignalP program (www.cbs.dtu.dk/services/SignalP). Theoretical isoelectric point (pI) and molecular weight (MW) were predicted by ScanSite PI/Mw (http://scansite.mit.edu/calc_mw_pi.html). Functional motifs were identified through the PROSITE database (http://prosite.expasy.org/). Potential protein phosphorylation sites were predicted by using NetPhos (http://www.cbs.dtu.dk/services/NetPhos/) [33], and transcription regulatory elements were identified using the Transcription Element Search System (TESS; http://www.cbil.upenn.edu/cgi-bin/tess/tess).

The upstream regions of *MfPG* genes were analyzed by the TESS program using the standard filters. Only domain sequences in the MFPG promoters resulting from this analysis having La/ parameter values of 2.0 and La parameter values higher than 12 were considered to be potential transcription factor binding sites. Multiple sequence alignments were performed using ClustalX 1.81 software (http://www.clustal.org/). Phylogenetic tree was constructed by the neighbor-joining methods with the PAUP*4.0β10 software (Sinauer Associates, Sunderland, MA, USA). The confidence values of the branches were determined by bootstrap analysis with 1000 replicates.

## Supporting Information

S1 Fig Phylogenetic analysis revealing the relationship of MFPGs to polygalacturonase (PG) family of *Sclerotinia sclerotiorum* and *Botrytis cinerea*. PGs from *S*. *sclerotiorum* and *B*. *cinerea* are abbreviated as SSPG and BCPG, respectively. The tree was constructed by aligning multiple sequence using cluster algorithms with the neighbor-joining clustering method. The bootstrap values represent the percentage of occurrence obtained from analysis of 1000 random samples.
(TIF)

S2 Fig Predicted transcriptional binding domains in the promoter region of *MfPGs*. The conserved binding domains were identified using a Transcription Element Search System (TESS) program with the standard filters. Abbreviations: TBP, TATA box binding protein; CRE1, carbon catabolite repressor; AreA/NIT2, activator of nitrogen-regulated genes. N, sense strain; R, antisense strain.
(TIF)

S3 Fig Quantitative reverse transcriptase PCR analysis of *MfPGs* expression in *M. fructicola* cultured in medium adjusted to various pHs. The ΔCt values (MfPG Ct– β-tubulin Ct) from three replicates in each experiment were calculated. The means of ΔCt from two independent experiments were used to calculate the –ΔΔCt value using the comparative C_T_ method. The ΔCt values representing the expression levels of *MfPGs* in response to different pHs were compared to that of the treatment at pH 4.0.
(TIF)

S4 Fig Southern blot analysis of *MfCUT1* copy number in *M*. *fructicola* genomic DNA. Genomic DNA was digested with *Bam*HI (B), *Hind*III (H) and *Xho*I (X), and hybridized with probe corresponding to *MFPG1* ORF. DNA size standards (kb) are indicated on the left.
(TIF)

S5 Fig Map of the transformation construct pUCATPH-MFPG1 (A) and Southern blot analysis of transformants (B). (A) A functional copy of *MfPG1* under its own promoter was cloned into pUCATPH carrying a hygromycin resistant gene cassette at *Sac*I site. (B) Fungal genomic DNA was isolated from the wild-type (M1) strain and seven transformants (5–1, 3–2, 4–1, 4–3, 5–2, 6–3 and 8–2) acquiring pUCATPH-MFPG1. Fungal DNA (10 μg) was digested with *Bam*HI, electrophoresed, blotted onto a nylon membrane and hybridized with an *MfPG1* DNA probe.
(TIF)

S6 Fig Polygalacturonase activity of the wild-type strain (M1) and the *MfPG1*-overexpressing strains (4–1 and 5–1). (TIF)

S7 Fig Reactive oxygen species accumulation on rose petals inoculated with the wild-type (M1) strain and *MfPG1*-overexpressing strains (5–1 and 8–2). Reactive oxygen species was detected with the oxidatively active fluorescent dye 2,7-dichlorofluorescin diacetate (DCFH-DA) 6 h post inoculation. The mock controls were treated with water only.
(TIF)

S1 Table Characteristics of endopolygalacturonase coding genes identified in *Monilina fructicola*. (DOCX)

S2 Table Comparative C_T_ method analysis for relative gene expression of *MfPG1*, *MfPG2*, *MfPG3*, *MfPG5* and *MfPG6* of *M*. *fructicola* grown in pectin medium at pH 4.0 for 24 h. (DOCX)

S3 Table Comparative C_T_ method analysis for relative gene expression of *MfPG1*, *MfPG2*, *MfPG3*, *MfPG5* and *MfPG6* of *M*. *fructicola* during pathogenesis on peach petal. (DOCX)

S4 Table Expression of *MfPG1*, *MfPG2*, *MfPG3*, *MfPG5* and *MfPG6* in the wild-type and the *MfPG1*-overexpressing strain 4–1 *in planta*. (DOCX)

S5 Table Oligonucleotide primers used in this study. (DOCX)
