# Supplementary figures and images for: Expression of Five Endopolygalacturonase Genes and Demonstration that MfPG1 Overexpression Diminishes Virulence in the Brown Rot Pathogen Monilinia fructicola
Source: PLoS One. 2015 Jun 29;10(6):e0132012. doi: 10.1371/journal.pone.0132012 (PMC4488289; doi:10.1371/journal.pone.0132012)

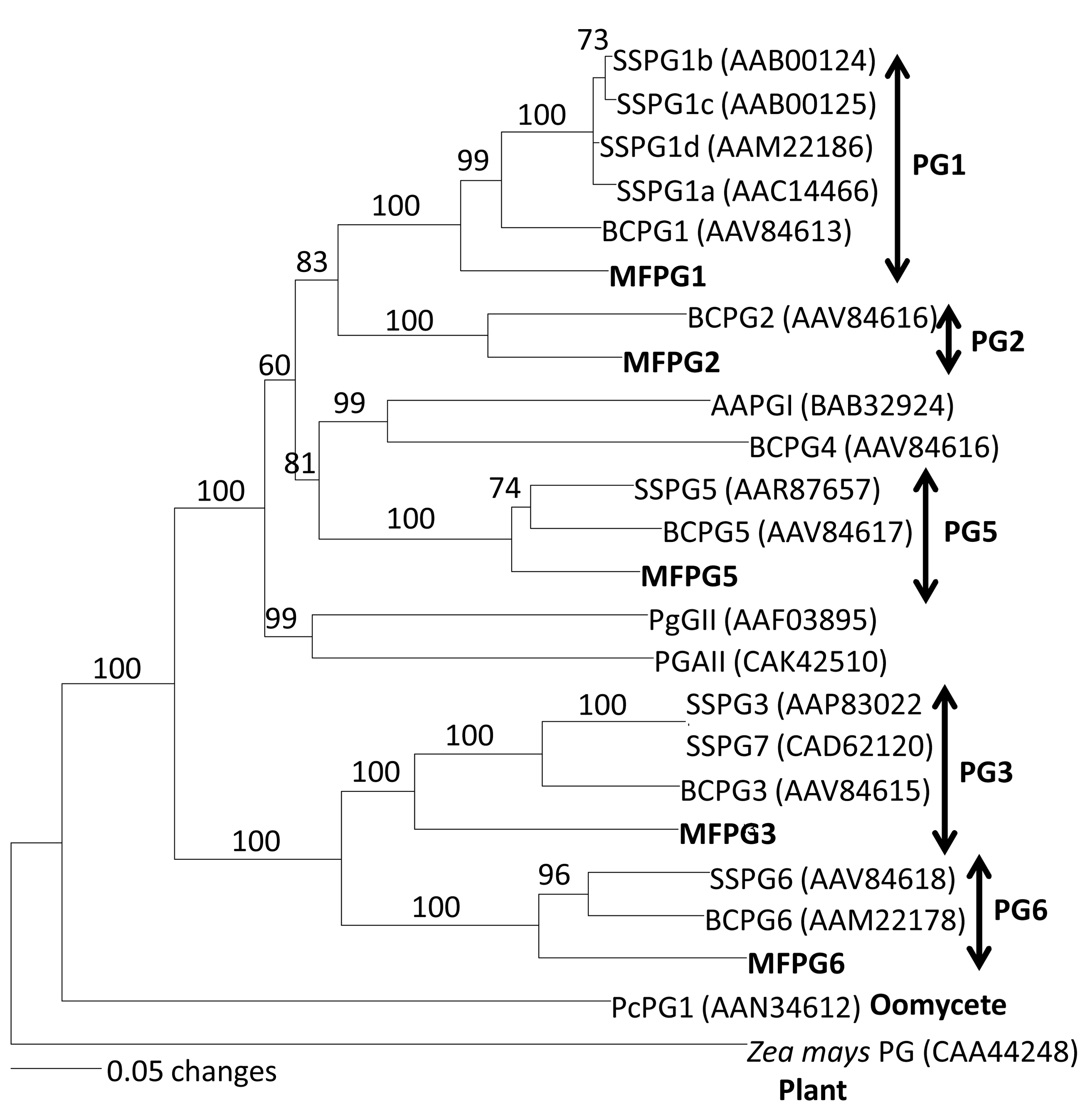

Supplement: S1 Fig — PGs from S. sclerotiorum and B. cinerea are abbreviated as SSPG and BCPG, respectively. The tree was constructed by aligning multiple sequence using cluster algorithms with the neighbor-joining clustering method. The bootstrap values represent the percentage of occurrence obtained from analysis of 1000 random samples. (TIF) [file pone.0132012.s001.tif]

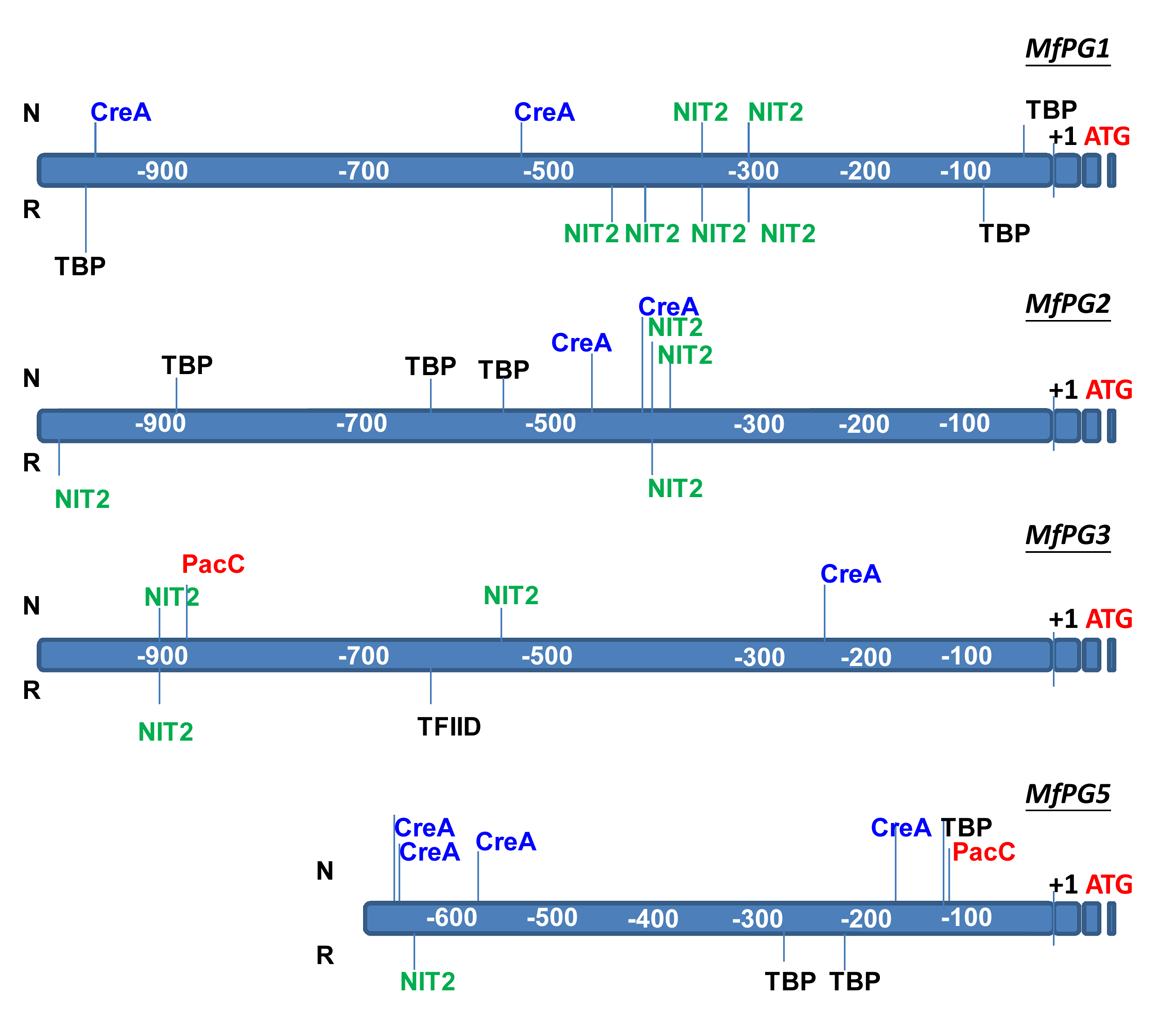

Supplement: S2 Fig — The conserved binding domains were identified using a Transcription Element Search System (TESS) program with the standard filters. Abbreviations: TBP, TATA box binding protein; CRE1, carbon catabolite repressor; AreA/NIT2, activator of nitrogen-regulated genes. N, sense strain; R, antisense strain. (TIF) [file pone.0132012.s002.tif]

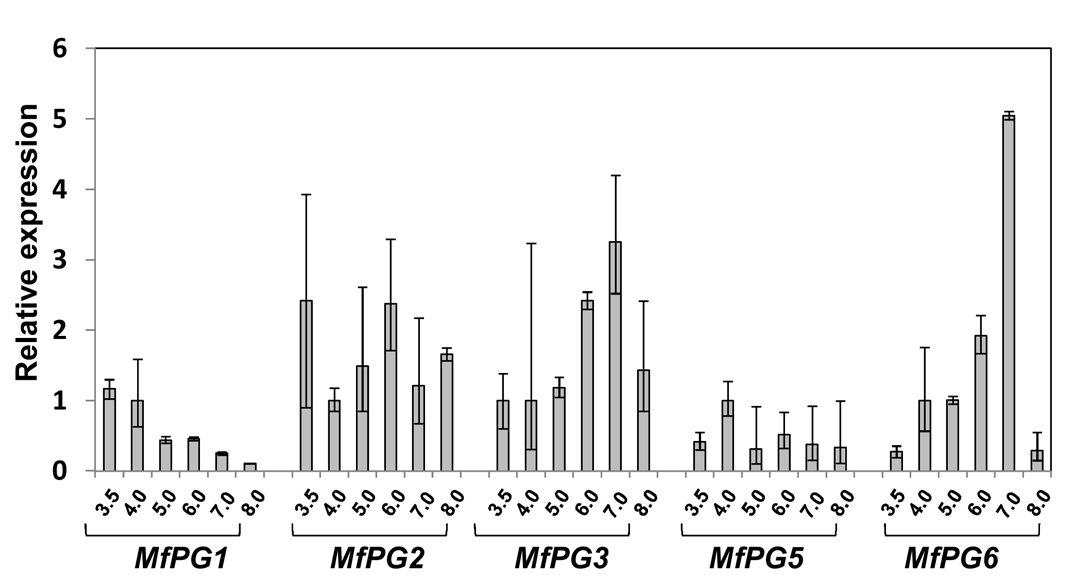

Supplement: S3 Fig — The ΔCt values (MfPG Ct– β-tubulin Ct) from three replicates in each experiment were calculated. The means of ΔCt from two independent experiments were used to calculate the –ΔΔCt value using the comparative CT method. The ΔCt values representing the expression levels of MfPGs in response to different pHs were compared to that of the treatment at pH 4.0. (TIF) [file pone.0132012.s003.tif]

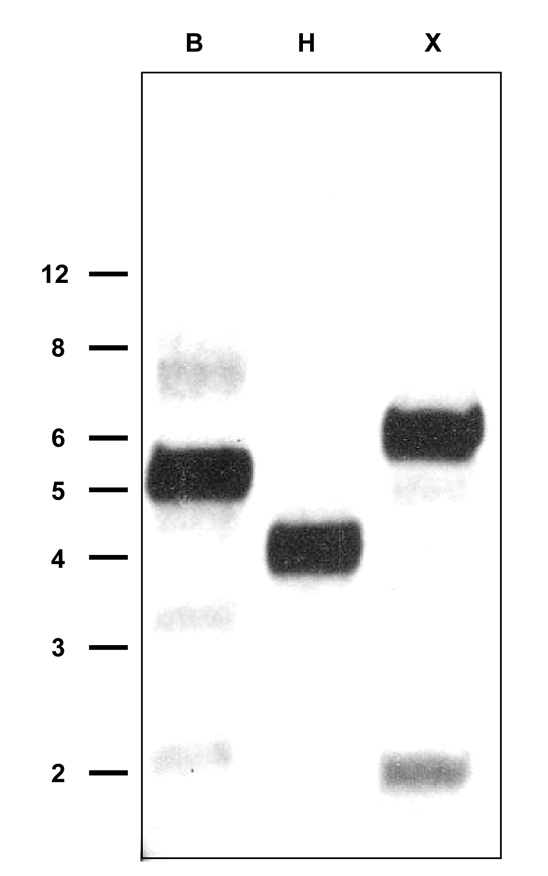

Supplement: S4 Fig — Genomic DNA was digested with BamHI (B), HindIII (H) and XhoI (X), and hybridized with probe corresponding to MFPG1 ORF. DNA size standards (kb) are indicated on the left. (TIF) [file pone.0132012.s004.tif]

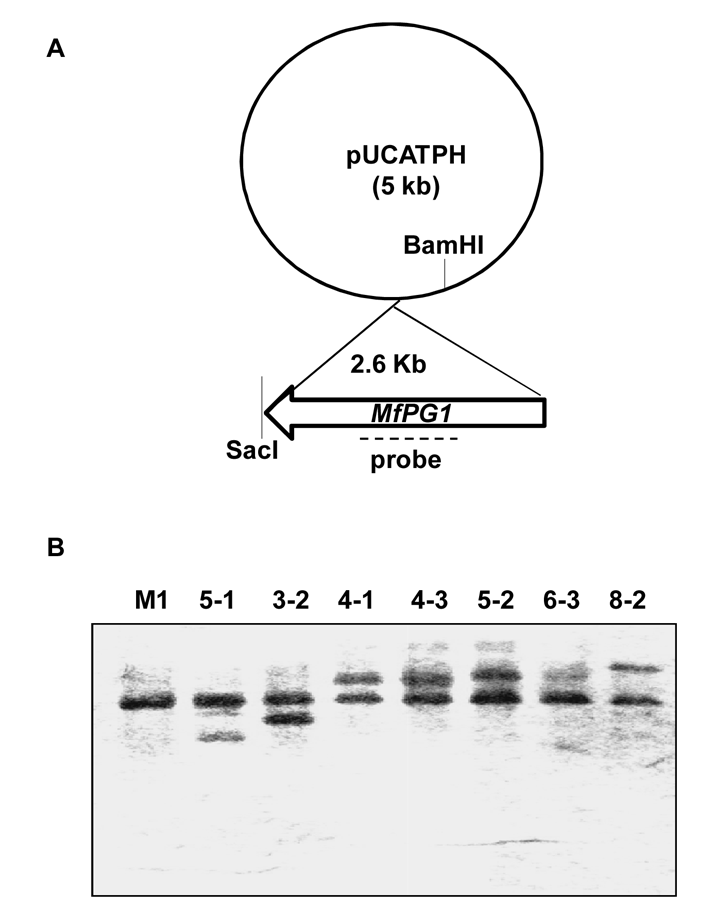

Supplement: S5 Fig — (A) A functional copy of MfPG1 under its own promoter was cloned into pUCATPH carrying a hygromycin resistant gene cassette at SacI site. (B) Fungal genomic DNA was isolated from the wild-type (M1) strain and seven transformants (5–1, 3–2, 4–1, 4–3, 5–2, 6–3 and 8–2) acquiring pUCATPH-MFPG1. Fungal DNA (10 μg) was digested with BamHI, electrophoresed, blotted onto a nylon membrane and hybridized with an MfPG1 DNA probe. (TIF) [file pone.0132012.s005.tif]

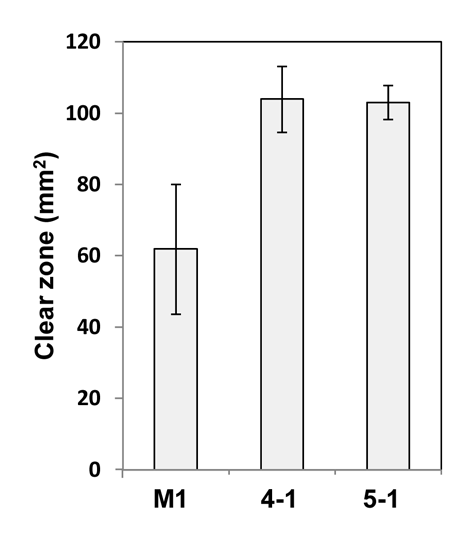

Supplement: S6 Fig — (TIF) [file pone.0132012.s006.tif]

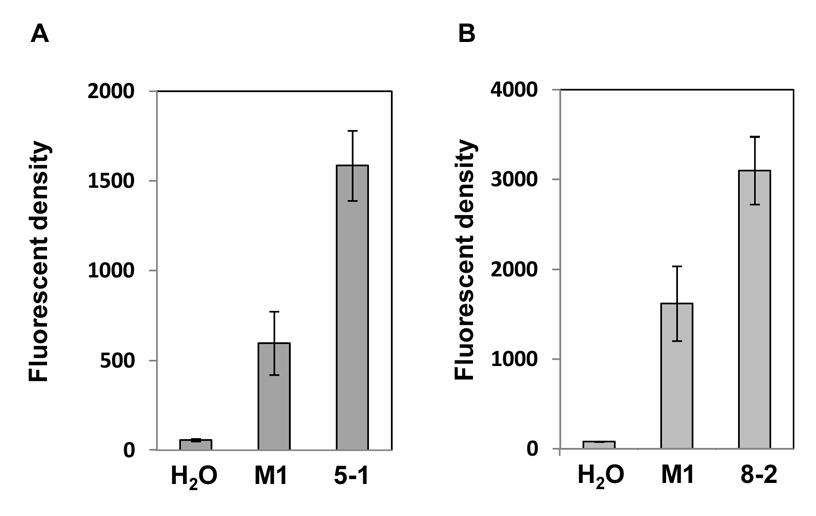

Supplement: S7 Fig — Reactive oxygen species was detected with the oxidatively active fluorescent dye 2,7-dichlorofluorescin diacetate (DCFH-DA) 6 h post inoculation. The mock controls were treated with water only. (TIF) [file pone.0132012.s007.tif]
