# Supplementary material for: Expression of Five Endopolygalacturonase Genes and Demonstration that MfPG1 Overexpression Diminishes Virulence in the Brown Rot Pathogen Monilinia fructicola
Source: PLoS One. 2015 Jun 29;10(6):e0132012. doi: 10.1371/journal.pone.0132012 (PMC4488289; doi:10.1371/journal.pone.0132012)
Supplement: S1 Table — (DOCX) [file pone.0132012.s008.docx]

**S1 Table.** Characteristics of endopolygalacturonase coding genes identified in *Monilina fructicola.*

| Gene | ORF | cDNA | Size of intron (bp) | Total a.a. | a.a. No. for signal peptide | Molecular weight of mature protein (kDa) | Theoretical isoelectric point (pI)^a^ | upstream flanking regions (bp)^b^ | downstream flanking regions (bp)^b^ |
| --- | --- | --- | --- | --- | --- | --- | --- | --- | --- |
| *MfPG1* | 1098 | 1098 | 0 | 365 | 20 | 34.8 | 9.02 | 1931 | 2562 |
| *MfPG2* | 1272 | 1107 | 62, 51, 52 | 368 | 19 | 35.0 | 8.90 | 1193 | 1145 |
| *MfPG3*^c^ | 1595 | 1533 | 62 | 510 | 16 | 50.1 | 9.12 | 1779 | 345 |
| *MfPG5* | 1391 | 1167 | 52, 51, 61, 60 | 388 | 19 | 37.1 | 4.76 | 687 | 1899 |
| *MfPG6*^c^ | 1215 | 1113 | 53, 49 | 370 | 21 | 36.7 | 5.37 | 60 | 522 |

^a^ Molecular weight and theoretical isoelectric point were predicted with the mature protein without post-translation modification..

^b^ Cloned *MfPGs* flanking sequence.

^c^ The presence of introns was predicted based on deduced amino acid sequences
