# Supplementary material for: Expression of Five Endopolygalacturonase Genes and Demonstration that MfPG1 Overexpression Diminishes Virulence in the Brown Rot Pathogen Monilinia fructicola
Source: PLoS One. 2015 Jun 29;10(6):e0132012. doi: 10.1371/journal.pone.0132012 (PMC4488289; doi:10.1371/journal.pone.0132012)
Supplement: S2 Table — (DOCX) [file pone.0132012.s009.docx]

**S2 Table.** Comparative C_T_ method analysis for relative gene expression of *MfPG1*, *MfPG2*, *MfPG3*, *MfPG5* and *MfPG6* of *Monilinia fructicola* grown in pectin medium at pH 4.0 for 24 h.

| Gene | ΔCt  (MfPG - tubulin) | SD of ΔCt ^a^ | -ΔΔCt | Fold difference of each *MfPG* relative to *MfPG2* ^b^ |
| --- | --- | --- | --- | --- |
| *MfPG1* | - 2.57 | 0.67 | 18.05 | 270762  (170247 – 430622) |
| *MfPG2* | 15.48 | 0.24 | 0.01 | 1  (0.85-1.18) |
| *MfPG3* | 9.60 | 1.69 | 5.89 | 59  (18-191) |
| *MfPG5* | 4.34 | 0.35 | 11.14 | 2259  (1771-2882) |
| *MfPG6* | 2.88 | 0.82 | 12.60 | 6194  (3519-10901) |

^a^ SD, Standard error for ΔCt (MfPG - tubulin) from three independent reactions.

^b^ The relative expression level of *MfPGs* was presented with fold difference by comparing to the expression level of *MfPG2*. Fold difference in each gene was indicated with average of three independent reactions. The lowest and highest fold difference value obtained was shown in parentheses.
