# Supplementary material for: Expression of Five Endopolygalacturonase Genes and Demonstration that MfPG1 Overexpression Diminishes Virulence in the Brown Rot Pathogen Monilinia fructicola
Source: PLoS One. 2015 Jun 29;10(6):e0132012. doi: 10.1371/journal.pone.0132012 (PMC4488289; doi:10.1371/journal.pone.0132012)
Supplement: S3 Table — (DOCX) [file pone.0132012.s010.docx]

**S3 Table.** Comparative C_T_ method analysis for relative gene expression of *MfPG1*, *MfPG2*, *MfPG3*, *MfPG5* and *MfPG6* of *Monilinia fructicola* during pathogenesis on peach petal.

| HPI^a^ | *MfPG* | ΔCt  (MfPG - tubulin) | SD of ΔCt ^b^ | -ΔΔCt | Fold difference relative to *MfPG6*^c^ |
| --- | --- | --- | --- | --- | --- |
| 0 | 1 | 2.52 | 0.27 | 3.50 | 11.29 (9.36 -13.61 ) |
|  | 2 | 4.76 | 0.10 | 1.26 | 2.40 (2.25-2.56) |
|  | 3 | 7.62 | 0.49 | - 1.60 | 0.33 (0.23-0.47) |
|  | 5 | 7.02 | 0.46 | - 1.00 | 0.50 (0.36 - 0.69) |
|  | 6 | 6.02 | 0.29 | 0.00 | 1.00 (0.81 - 1.22) |
|  | | | | | |
| 6 | 1 | - 0.31 | 0.28 | 4.67 | 25.52 (21.04 – 30.94) |
|  | 2 | 2.21 | 0.29 | 2.15 | 4.45 (3.65 – 5.42) |
|  | 3 | 5.28 | 0.23 | - 0.92 | 0.53 (0.45 - 0.62) |
|  | 5 | 6.11 | 0.33 | - 1.75 | 0.30 (0.24 - 0.37) |
|  | 6 | 4.36 | 0.23 | 0.00 | 1.00 (0.85 - 1.18) |
|  | | | | | |
| 10 | 1 | - 0.70 | 0.26 | 5.38 | 41.55 (34.72 – 49.72) |
|  | 2 | 1.61 | 0.33 | 3.07 | 8.40 (6.67 – 10.58) |
|  | 3 | 4.45 | 0.37 | 0.23 | 1.17 (0.90 - 1.52) |
|  | 5 | 6.88 | 0.13 | - 2.20 | 0.22 (0.20 - 0.24) |
|  | 6 | 4.68 | 0.18 | 0.00 | 1.00 (0.88 - 1.13) |
|  | | | | | |
| 28 | 1 | - 1.39 | 0.36 | 6.20 | 73.69 (57.27 – 94.80) |
|  | 2 | - 0.17 | 0.29 | 4.98 | 31.56 (25.82 – 38.57) |
|  | 3 | 7.57 | 0.30 | - 2.76 | 0.15 (0.12 - 0.18) |
|  | 5 | 5.24 | 0.29 | - 0.43 | 0.74 (0.61 - 0.91) |
|  | 6 | 4.81 | 0.14 | 0.00 | 1.00 (0.91 - 1.10) |
|  | | | | | |
| 48 | 1 | - 0.86 | 0.14 | 8.28 | 310.83 (281.47 – 343.26) |
|  | 2 | 3.17 | 0.41 | 4.25 | 18.98 (14.32 – 25.17) |
|  | 3 | 8.22 | 0.38 | - 0.80 | 0.57 (0.44 - 0.75) |
|  | 5 | 7.70 | 0.42 | - 0.28 | 0.82 (0.61 – 1.10) |
|  | 6 | 7.42 | 0.20 | 0.00 | 1.00 (0.87 - 1.15) |

^a^ HPI, hour post-inoculation.

^b^ SD, Standard error for ΔCt (MfPG - tubulin) from three independent reactions.

^c^ The relative expression level of *MfPGs* was presented with fold difference by normalizing to the expression level of *MfPG6* at each time point. Fold difference in each gene was indicated with average of three independent reactions. The lowest and highest fold difference value obtained was shown in parentheses.
