# Supplementary material for: Expression of Five Endopolygalacturonase Genes and Demonstration that MfPG1 Overexpression Diminishes Virulence in the Brown Rot Pathogen Monilinia fructicola
Source: PLoS One. 2015 Jun 29;10(6):e0132012. doi: 10.1371/journal.pone.0132012 (PMC4488289; doi:10.1371/journal.pone.0132012)
Supplement: S4 Table — (DOCX) [file pone.0132012.s011.docx]

**S4 Table.** Expression of *MfPG1, MfPG2, MfPG3, MfPG5* and *MfPG6* in the wild-type and the *MfPG1*-overexpressing strain 4-1 *in planta.*

| Hour post-inoculation | Fold difference of each *MfPG* gene in *MfPG1*-overexpressing strain 4-1 relative to wild type strain ^a^ | | | | |
| --- | --- | --- | --- | --- | --- |
|  | *MfPG1* | *MfPG2* | *MfPG3* | *MfPG5* | *MfPG6* |
| 5 | 1.98  (1.84-2.14) | 0.75  (0.72-0.78) | 0.66  (0.31-1.40) | ND | 1.20  (1.12-1.29) |
| 24 | 3.18  (2.95-3.44) | 4.20  (4.05-4.35) | 0.32  (0.15-0.68) | 3.21  (2.47-4.16) | 0.92  (0.86-0.99) |

^a^ The relative expression levels were calculated using a comparative C_T_ method. Fold difference in each gene was indicated with average of three independent reactions. The lowest and highest fold difference value obtained was shown in parentheses.
