# Supplementary material for: Expression of Five Endopolygalacturonase Genes and Demonstration that MfPG1 Overexpression Diminishes Virulence in the Brown Rot Pathogen Monilinia fructicola
Source: PLoS One. 2015 Jun 29;10(6):e0132012. doi: 10.1371/journal.pone.0132012 (PMC4488289; doi:10.1371/journal.pone.0132012)
Supplement: S5 Table — (DOCX) [file pone.0132012.s012.docx]

**S5 Table.** Oligonucleotide primers use in this study.

| Primers | Sequences (5’-3’) | Purpose |
| --- | --- | --- |
| MFPG1-F2 MFPG1-R1 | gatgactgtctcgctatcaactccgg  acttgacaccagatgggagaccagc | *MfPG1* cloning |
| MFPG1-CF1  MFPG1-CR1 | ggttcaaatcgtttcatcgg  aacacttgacaccagatggg | *MfPG1* cDNA cloning and probe for DNA/RNA blot analysis. |
| MFPGDP-F2  MFPGDP-R2 | aaycargatgaytgystngc  acrccrttdgtsggktydcc | Degenerate primer pairs designed for *MfPG*s cloning |
| MFPG2-F1  MFPG2-R1 | ggcaaagtmaagcctaagttc  ttccaagtccagccwgagc | *MfPG2* cloning |
| MFPG2-CF1  MFPG2-CR1 | gttcaattgtcaactctcgttacc  ttagcaggaagctccggatg | *MfPG2* cDNA cloning and probe for RNA bolt |
| MFPG2-IF1  MFPG2-IR1 | ggtgttgtgaccaaggttgc  tggtagcccaactggcatac | *MfPG2* flanking sequence cloning with inverse PCR |
| MFPGG2-IF2  MFPG2-IR2 | attgaactggggagttgagg  ccgtctctggtggtcaaaac | *MfPG2* flanking sequence cloning with nested inverse PCR |
| MFPG3-F1  MFPG3-R1 | ggcaaagtmaagcctaagttc  ttccaagtccagccwgagc | *MfPG3* cloning |
| MFPG3-CF1  MFPG3-CR1 | gcgttctgcgattattctcg  ctatgctggacatccggtag | *MfPG3* cDNA cloning and probe for RNA bolt |
| MFPG3-IF1  MFPG3-IR1 | gtccatgtcctcctgagca  tctaccatcaccaacagtcg | *MfPG3* flanking sequence cloning with inverse PCR |
| MFPG3-IF2  MFPG3-IR2 | aacagcaacgcaatcatcttg  cggaaacccgacaaatgg | *MfPG3* flanking sequence cloning with nested inverse PCR |
| MFPG5-F1  MFPG5-R1 | ggcaaagtmaagcctaagttc  ttccaagtccagccwgagc | *MfPG5*cloning |
| MFPG5-CF1  MFPG5-CR1 | gttcagctttctgcctatcttc  ctataatttagcaagcggcacc | *MfPG5* cDNA cloning and probe for RNA bolt |
| MFPG5-IF1  MFPG5-IR1 | gtccatgtcctcctgagca  tctaccatcaccaacagtcg | *MfPG5* flanking sequence cloning with inverse PCR |
| MFPG5-IF2  MFPG5-IR2 | aacagcaacgcaatcatcttg  cggaaacccgacaaatgg | *MfPG5* flanking sequence cloning with nested inverse PCR |
| MFPG6-DF1  MFPG6-DR1 | ttcccaaatcgctccttgtgttgc  ccwgtratyttractccmgagaa | Degenerate primer pairs designed for *MfPG6* cloning |
| MFPG6QF1  MFPG6R1 | ctactacgtgctatgtggtc  gcaaatgtgactgtagtgcc | *MfPG6* flanking sequence cloning with inverse PCR |
| MFPG1-SmaI MFPG1-SacI | tcccccgggggacctttgcacaagccagattt  ggcgagctcgcccgcatttagaaagggcagag | MfPG1 overexpression construct preparation |
| MFPG-EcoRI-901 MFPG-SpeI-3026 | gcgcaagcttccctcgccctcggtcccctttttc ggccactagtggagcctgcaacttgcttgacgaaa | To generate MFPG1-GUS fusion |
| EcoRI-MFPG67  ApaI-MFPG2480 | cggcgaattcctcagatagatgcattagctcccctttg  gcgcgggcccgtgtccaccaccggcatcaccagcag | *MfPG1* replacement construct preparation |
| XhoI-MFPG2725  SacI-MFPG5479 | gcgcctcgagccatctccggtgctactggaaccgtc  gccggagctcgctttgccg gttgagcagcatgatgag | *MfPG1* replacement construct preparation |
| nptII-Apa  nptII-Xho | gcgcgggccctcgatctagtaacatagatgacaccgc  gccgagatctgggtttctggagtttaatgagctaag | *MfPG1* replacement construct preparation |
| Mfpg-1  NptII-2 | gggggagagtagcatgatga  agacaatcggctgctctgat | Split marker preparation |
| Mfpg-2  NptII-1 | ccatttcaacaccgtcactg  aatatcacgggtagccaacg | Split marker preparation |
| NptII-Fs2  NptII-Rs2 | agaggctattcggctatgac  ctatttgctcttggacgttg | Homologous recombination confirmation |
| TUBQF  TUBQR | ttcttgatgttgttcgtcgtg  gggtgatttggaaaccttga | qPCR for β-tubulin, 63 bp |
| MFPG1QF  MFPG1QR | tgtcaacgttaccggtggt  ttgacaccagatgggagacc | qPCR for MfPG1, 60 bp, |
| MFPG2QF  MFPG2QR | actgctaaggcccaaaagg  tggaacagtaatgccgttga | qPCR for MfPG2, 60 bp |
| MFPG3QF  MFPG3QR | atggtagaggtatcacgatcactg  cggcaccatttccttcaat | qPCR for MfPG3, 60 bp |
| MFPG5QF  MFPG5QR | ggttccaacggaggaaagac  cgaattgatcaagttgtgtgc | qPCR for MfPG5, 60 bp |
| MFPG6QF3  MFPG6QR3 | ggcccgttcatcttttcac  ccctgctgtgttgttgagag | qPCR for MfPG6, 77 bp |
